# NLRC5, at the Heart of Antigen Presentation

**DOI:** 10.3389/fimmu.2013.00397

**Published:** 2013-11-22

**Authors:** Andreas Neerincx, Wilson Castro, Greta Guarda, Thomas A. Kufer

**Affiliations:** ^1^Institute for Medical Microbiology, Immunology and Hygiene, University of Cologne, Cologne, Germany; ^2^Department of Biochemistry, University of Lausanne, Epalinges, Switzerland

**Keywords:** NLRC5, antigen presentation, transcription, innate signaling, NLR, MHC class I

## Abstract

Nucleotide-binding domain and leucine-rich repeat containing receptors (NLRs) are intracellular proteins mainly involved in pathogen recognition, inflammatory responses, and cell death. Until recently, the function of the family member NLR caspase recruitment domain (CARD) containing 5 (NLRC5) has been a matter of debate. It is now clear that NLRC5 acts as a transcriptional regulator of the major-histocompatibility complex class I. In this review we detail the development of our understanding of NLRC5 function, discussing both the accepted and the controversial aspects of NLRC5 activity. We give insight into the molecular mechanisms, and the potential implications, of NLRC5 function in health and disease.

## Introduction

**Figure 1:**
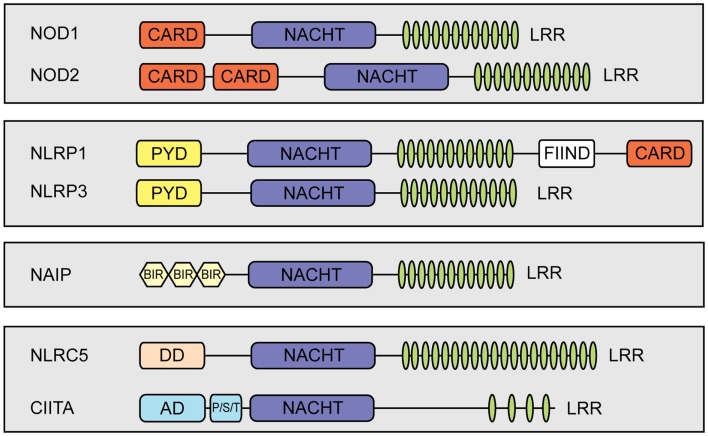
**Schematic representation of members of the mammalian NLR-family**. NLRs share a tripartite domain architecture and can be subdivided based on the identity of their N-terminal effector domain which links to different cellular signaling pathways. All NLRs contain a central nucleotide-binding domain (NACHT) that mediates oligomerization. In addition, most NLRs contain putative ligand-sensing leucine-rich repeats (LRRs) and a variable N-terminal effector domain. The effector domain can be a pyrin domain (PYD), a caspase recruiting domain (CARD), or a baculovirus inhibitor of apoptosis repeat (BIR). Additional abbreviations: FIIND, function to find; DD, death-domain different from a typical CARD and PYD; AD, activation domain; P/S/T, proline/serine/threonine-rich protein domain.

Nucleotide-binding domain and leucine-rich repeat (LRR) containing proteins (NLRs) play pivotal roles as intracellular pattern recognition receptors (PRRs) mediating detection of invading pathogens and triggering innate immune responses. Most NLRs are involved in either NF-κB signaling or interleukin (IL)-1β and IL-18 processing (1, 2). Nonetheless, functions beyond pathogen recognition have been reported for several NLRs [reviewed in Ref. (3)]. In mammals, NLRs are a large protein family, including 22 members in humans (4). They have a common tripartite structure, which consists of an N-terminal effector domain, a central nucleotide-binding domain and a C-terminal LRR-containing region [reviewed in Ref. (3, 5)]. The N-terminal effector domain of NLR proteins is in most cases either a caspase recruitment domain (CARD) or a pyrin domain that link to different signaling pathways [reviewed in Ref. (6, 7)] (Figure 1).

In this review, we focus on the NLR CARD containing 5 (NLRC5), a recently characterized family member involved in the regulation of major-histocompatibility complex (MHC) class I transcription. In particular, we will discuss the current understanding of NLRC5 expression patterns; the function of NLRC5 *in vitro* and *in vivo*; and how NLRC5 is implicated in immunological function and disease.

## The MHC Class II Transcriptional Activator: A Paradigm for NLRC5 Function

The best example of an NLR protein that does not function as a PRR is the MHC class II transcriptional activator (CIITA). In contrast to other NLR proteins, CIITA harbors a unique N-terminal transcription activation domain (AD) followed by a proline/serine/threonine-rich region (P/S/T) [reviewed in Ref. (8)]. CIITA was originally discovered through an expression cloning approach using an MHC class II deficient clone of the Burkitt Lymphoma B cell line Raji (9–12). Steimle and colleagues identified a cDNA that complemented MHC class II expression, and termed the encoded protein CIITA (12).

**Figure 2:**
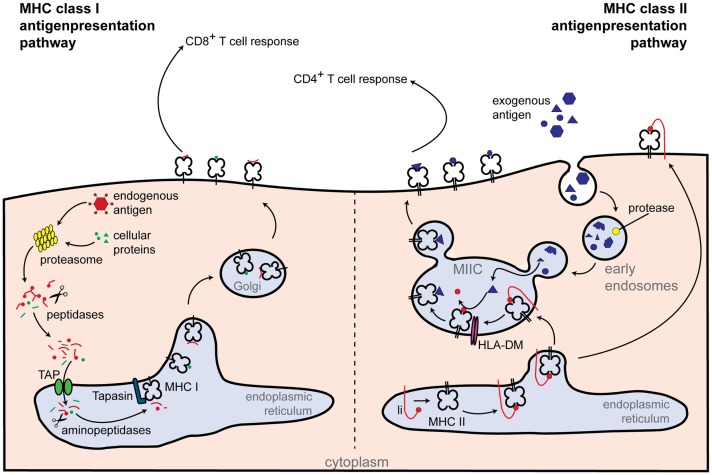
**Classical routes of antigen presentation by MHC class I and II molecules**. Class I antigen presentation (left-hand side): proteasomes generate peptides from all proteins present within the cell. Peptide fragments are transported to the endoplasmic reticulum, where they are further trimmed by aminopeptidases and loaded onto the MHC class I molecule. MHC class I-loaded complexes are transported to the cell surface, where they are presented to CD8^+^ T cells. Class II antigen presentation (right-hand side): extracellular antigens are taken up within phagosomes by APCs. Phagosomes fuse with lysosomes, which contain proteolytic enzymes that cleave the phagocytosed proteins into small peptides. Newly synthesized MHC class II molecules from the endoplasmic reticulum are delivered to the phagolysosomes and loaded with peptide. Peptide-loaded MHC class II complexes are transported to the cell surface, allowing antigen presentation to CD4^+^ T cells. Additional abbreviations: Ii, MHC class II-associated invariant chain; MIIC, MHC class II compartment; TAP, transporter associated with antigen processing.

Major-histocompatibility complex class II molecules are expressed constitutively on professional antigen presenting cells (APCs) and display antigens of exogenous origin. Phagocytosed antigens undergo endosomal degradation and are loaded onto MHC class II molecules in the so-called “MHC class II compartment.” Peptide-MHC class II complexes are then transported to the cell surface, where foreign antigens activate CD4^+^ T cells [reviewed in Ref. (13)] (Figure 2). Not surprisingly, CIITA loss of function mutants are associated with a severe immunodeficiency called bare lymphocyte syndrome (BLS). This condition is characterized by a lack of MHC class II expression, deficiency in helper T cells, and impaired humoral and cytotoxic responses (12).

In humans, CIITA transcription is tightly regulated and can be induced by the differential use of four alternative promoters, pI–pIV. However, the relevance of pII, which is used at low rate, remains unclear and only pI, pIII, and pIV are conserved in mice (14). Each promoter initiates transcription at unique sites, resulting in four isoforms of CIITA that differ in their N-terminus. pIII is mainly used in B cells, activated human T cells, and plasmacytoid dendritic cells (pDCs) (8, 14). By contrast, pI is active in DCs and results in a transcript that encodes an N-terminal CARD-domain. This isoform is the most efficient at inducing MHC class II expression *in vitro* (15), though its physiological role remains elusive (16). Interferon (IFN)-γ leads to high induction of CIITA expression, mainly through activation of pIV (14, 17).

**Figure 3:**
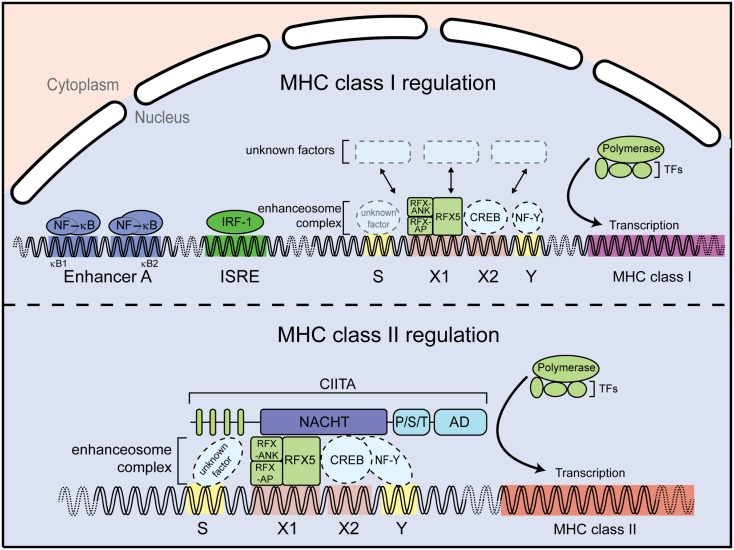
**Major-histocompatibility complex class I and II promoters**. MHC class I gene regulation (upper panel). Expression of MHC class I genes is regulated by several elements in the promoter region. For instance, the enhancer A sequence allows binding of the transcription factor NF-κB; the interferon stimulated response element (ISRE) serves as an active site for interferon regulator factor 1 (IRF1). In addition, a conserved SXY module is found in the proximal MHC class I gene promoters. MHC class II gene regulation (lower panel). The MHC class II gene contains an SXY module that facilitates assembly of the so-called enhanceosome. The enhanceosome is a protein complex containing regulatory factor X (RFX) proteins, cAMP response element-binding protein (CREB), and the nuclear factors Y (NF-Y). The assembly of this protein complex constitutes a platform for the recruitment the class II transactivator CIITA. Additional abbreviation: RFX-AP, RFX-associated protein; RFX-ANK, RFX-associated ankyrin-containing protein; TFs, transcription factors.

In addition to CIITA, MHC class II transcription requires the DNA-binding factors regulatory factor X (RFX) 5, RFX-AP, and RFX-ANK (or RFX-B) (18–21). These RFX proteins specifically bind to the X1-box inside the conserved SXY motif of the MHC class II promoters (22) (Figure 3). They assemble, together with X2BP (23, 24) and NF-Y factors (25, 26), into a DNA-binding platform known as the enhanceosome. CIITA itself does not directly bind DNA but rather uses the enhanceosome to dock to the MHC class II promoter to activate gene transcription by recruiting the general transcription machinery and histone-modifying enzymes [reviewed in Ref. (27, 28)] (Figure 3). CIITA not only governs the transcriptional regulation of the MHC class II α-chain and β-chain, but also regulates expression of other genes in the MHC class II locus, including the invariant chain (li), human leukocyte antigen (HLA)-DM, and HLA-DO. Interestingly, CIITA has also been reported to control expression of IL-4, Fas-ligand (CD95L), collagen A2, and some viral proteins [summarized in Ref. (29)].

By contrast to the presentation of exogenous antigens by MHC class II molecules to CD4^+^ T cells, endogenous peptides are presented by MHC class I to CD8^+^ T cells. Differently from MHC class II, MHC class I gene expression can be regulated by NF-κB and IFN-sensitive response element (ISRE) binding sites located within the promoter regions, which are absent in MHC class II promoters. In particular the classical MHC class I molecules HLA-A and HLA-B can be induced by NF-κB and are highly transactivated by IFN-γ (30, 31) (Figure 3). However, transcription of MHC class I genes, which are expressed in almost all nucleated cells, also occurs in the absence of NF-κB or interferon regulatory factor (IRF) signaling. MHC class I levels differ between tissues and cell types, indicative of a complex regulatory network (32). Interestingly, an SXY motif is also present in MHC class I promoter regions (33) (Figure 3) and several groups reported an involvement of CIITA in MHC class I expression *in vitro* via the enhanceosome (34–37). However, this could not be validated *in vivo*, as *Ciita*-deficient mice show normal MHC class I expression, suggesting the presence of additional factors driving basal MHC class I expression through the SXY regulatory region (38).

Intriguingly, alignment studies of the human NLR-family performed for the nucleotide-binding domain and the LRR region revealed a close homology between human CIITA and human NLRC5, suggesting that the latter might exhibit a function analogous to CIITA (39–42). Although, NLRC5 shows a typical tripartite NLR domain organization, it should be mentioned that it contains a rather unusual long LRRs and harbors a N-terminal effector domain with a predicted death-domain (DD) fold different from regular DD, DED, PYD, or CARD domains (39, 43, 44).

## NLRC5 Expression and Regulation

Recent insights into the expression of NLRC5 have shown constitutive levels in several tissues. mRNA is abundant in lymphoid organs such as lymph nodes and spleen, and – particularly in humans – in the lung and the intestinal tract. This implies a role for NLRC5 in the immune response and may suggest a specific role at mucosal surfaces (41, 43, 45). Taking a closer look at hematopoietic cells, high levels of *NLRC5* transcripts were observed in T and B cells, and lower levels in CD14^+^ leukocytes. A detailed analysis of NLRC5 expression at the protein level in murine tissues and primary hematopoietic cells confirmed these observations (46).

NLRC5 is induced by several stimuli including type I and II IFNs (41, 42, 46, 47). To better understand the transcriptional regulation of *NLRC5*, Kuenzel and co-workers analyzed the promoter region of the human *NLRC5* gene using a bioinformatic approach. They identified putative *cis*-elements that might regulate *NLRC5* expression (47). These regulatory elements consist of two signal transducer and activator of transcription (STAT) consensus-binding sites at position −1336 and −452 that, according to Genomatix, would be specific for the transcription factors STAT1 or STAT3 (48). A potential NF-κB consensus-binding site, partially overlapping with the STAT binding site at position −1336, was identified in the human promoter (47). Kuenzel and co-workers showed that IFN-γ-mediated *NLRC5* promoter transactivation was partially abrogated in the absence of either STAT binding site. Accordingly, in different hematopoietic mouse cells and human HeLa cells, NLRC5 expression was increased early upon treatment with both type I and II IFNs in a STAT1-dependent manner (42, 46). However, it is important to point out that an ISRE binding site, which can be bound by STAT and IRF complexes, has been predicted in the promoter of NLRC5 (44, 47). Therefore, IFN-mediated *NLRC5* induction could be dependent on this additional regulatory sequence. Thus, the importance of these predicted binding sites in NLRC5 maintenance or induction still awaits thorough characterization.

Innate immune stimuli such as polyinosinic-polycytidylic acid (Poly I:C) and lipopolysaccharide (LPS) efficiently induce NLRC5 expression in bone marrow-derived macrophages (BMDMs) through the autocrine effect of type I IFNs. This was demonstrated with the use of *Ifnar-* and *Stat1*-deficient cells (46). In addition the use of *Trif* (TIR-domain-containing adapter-inducing IFN-β)-deficient and *Myd88* (myeloid differentiation primary response gene 88)-deficient BMDMs showed that upregulation of NLRC5 by LPS was dependant on the toll-like receptor (TLR) 4 adaptor protein Trif (46). Moreover, inflammatory stimuli such as IFN-γ, poly I:C, or Sendai Virus (SeV), a virus inducing strong IFN responses, also enhanced NLRC5 expression in human primary dermal fibroblasts, the epithelial carcinoma cell line HeLa, and the colon carcinoma cell lines CaCo2 and HT29 (43, 47).

Since several isoforms have been described for *CIITA*, it is conceivable that *NLRC5* could also possess different mRNA variants. Indeed, six isoforms varying in their C-terminal LRR sequences have been reported in databases (www.uniprot.org; Q86WI3). Isoform 3, which is missing the entire LRR region, was found to be mainly expressed in CD8^+^ and CD4^+^ lymphocytes. Isoforms 4 and 5, which both lack exon 25, were detected at low levels in THP-1 cells (43). Whether these different NLRC5 isoforms have any biological relevance remains to be established. While for some NLRs, such as NOD1 and NOD2, the LRR region has been involved in sensing pathogen- or danger-associated molecular patterns (PAMPs or DAMPs) (49, 50), it can also have autoinhibitory functions (1). Thus the different isoforms of NLRC5 deserve further investigation, as they could either diversify its potential ligand binding ability or alter its autoregulatory activity.

## NLRC5 Controls Basal MHC Class I Gene Expression

Given the phylogenetic proximity between CIITA and NLRC5, Meissner and co-workers used overexpression and RNA interference (RNAi)-mediated knockdown to study the capacity of NLRC5 to activate MHC gene transcription (42). They found that NLRC5 overexpression induced classical MHC class I molecules in HEK293T and in Jurkat cells. This finding has been subsequently confirmed and extended to both primary cells and other cell lines (42, 46, 51–56). Interestingly, human NLRC5 is able to restore MHC class I expression in the murine melanoma cell line B16F10, which is defective for MHC class I expression (53). NLRC5 knockdown in THP-1 cells, poly I:C-treated Jurkat T cells, HeLa cells, and human dermal fibroblasts confirmed that NLRC5 contributes to MHC class I expression in these cells (42, 53). Moreover, HLA-B and NLRC5 expression correlate in several human tissues supporting the function of NLRC5 as a transactivator of MHC class I genes (53), although discordant results from lung tissue suggests the existence of additional control mechanisms.

Classical MHC class I molecules are highly polymorphic and consist of a heavy chain (encoded by the genes HLA-A, -B, and -C) and the β_2_-microglobulin chain. Peptide fragments presented in MHC class I mainly derive from cytosolic proteins degraded by the 26S proteasome which can be supported by additional subunits forming the so-called “immunoproteasome” (57). Peptides are then transported into the endoplasmic reticulum (ER) by the peptide transporter associated with antigen processing (TAP), in which the MHC class I molecules are present as membrane-bound proteins stabilized by a subset of chaperones. Following further aminopeptidase-mediated cleavage, peptides are loaded onto the MHC class I molecule by the peptide-loading complex, which includes TAP1, TAP2, and tapasin. Antigen-loaded MHC class I molecules are transported via the Golgi-network to the cell surface, where they are detected by specific receptors on CD8^+^ T cells, as depicted in Figure 2 [reviewed in Ref. (13)]. Interestingly, NLRC5 also controls the expression of MHC class I-related genes, such as β_2_-microglobulin, TAP1, and the immunoproteasome subunit low molecular mass poylpeptides 2 (LMP2) (42).

The forced expression of NLRC5 also leads to induction of the non-classical MHC class I genes HLA-E, F, G, less polymorphic molecules that are mainly involved in Natural Killer (NK) cell inhibition (42). Altogether, these data indicate NLRC5 to be a key player in the process of MHC class I-mediated antigen presentation.

## Molecular Aspects of NLRC5 Transcriptional Regulatory Function

### NLRC5 transcriptional regulatory activity depends on the enhanceosome

As discussed above, CIITA-dependent MHC class II activation requires the enhanceosome complex, which binds to the SXY motif of the MHC class II promoter region [reviewed in Ref. (8, 27)]. A similar region in the MHC class I promoter was shown to be important for enhanceosome-dependent MHC class I activation (36) (Figure 3). Importantly, NLRC5 has been shown to occupy the promoter of MHC class I genes in the region encompassing the SXY module by chromatin immunoprecipitation assays (42, 46). Mutation of the X1- and X2-motifs, as well as the absence of selected enhanceosome components, abolished NLRC5-dependent MHC class I activation, demonstrating that NLRC5-mediated MHC class I activation is dependent on components of the enhanceosome (53, 58). Consistent with this, ankyrin repeat dependent interaction of RFX-ANK and NLRC5 has been reported (58). In addition, Meissner and colleagues identified the S-motif as important for NLRC5-dependent, but not CIITA-dependent, transactivation (Figure 3) (58). The S-motif has been implicated in enhanceosome-dependent regulation of MHC transcription (59). However, its precise role in NLRC5-dependent gene regulation still needs to be established.

Altogether, these results reveal that NLRC5 exerts transcriptional regulation in an enhanceosome-dependent manner. A future challenge is to understand the epigenetic mechanisms underlying NLRC5-dependent transactivation of MHC class I gene promoters; the first insights into this important aspect are just starting to emerge, as recently reviewed (44).

### Molecular mechanisms of NLRC5 nuclear localization

The transcriptional function of NLRC5 requires an ability to shuttle into the nucleus. NLRC5 strongly accumulates in the nucleus upon inhibition of nuclear export, while at steady-state conditions only a minor portion is detectable within the nucleus (41, 42, 46, 47, 53). In contrast, CIITA is evenly distributed between cytoplasm and nucleus (60, 61). An N-terminally located nuclear localization sequence (NLS) is responsible for the nuclear shuttling of NLRC5 (42, 52, 53). However, this process is also reported to depend on the Walker A motif that constitutes the nucleotide-binding site of the central nucleoside triphosphatase (NTPase) domain (see Figure 1) (52, 53). Of note, disruption of the Walker A box in CIITA was also shown to diminish MHC transactivation and nuclear shuttling (62, 63). However, these results should be interpreted cautiously, as mutation of the Walker A site might affect overall protein structure.

NLRC5 and CIITA show close sequence homology in their LRR regions (40). However, the LRR regions differentially affect their subcellular localization. For CIITA it has been shown that several structural features of the LRRs are important for nuclear import (60, 61, 64). In contrast the LRR region of NLRC5 is dispensable for nuclear import, but appears to be involved in nuclear export. NLRC5 constructs lacking the LRR region showed nuclear localization even in the absence of blockage of nuclear export (52, 53).

Paradoxically, forced nuclear localization of NLRC5 (by fusion of a strong viral NLS) results in reduced MHC class I transactivation potential (52, 53). This could mean that the transcriptional activity of NLRC5 might depend on cytoplasmic modifications that cannot be carried out correctly if the protein only resides briefly in the cytoplasm. Of note, NLRC5 runs as a double band in SDS-gel electrophoresis, which might be indicative of such a posttranslational modification, although its nature remains to be established (46, 47). Alternatively, enhanced nuclear import of NLRC5 might interfere with the formation of NLRC5 protein–protein complexes in the cytosol that confer transcriptional activity upon translocation to the nucleus.

### Hints from NLRC5-analogous proteins in mammalian and plant cells

As discussed above, our understanding is that NLRC5, similarly to CIITA, is active without any DAMP or PAMP stimulus; its activity relying mainly on the expression level. This is in sharp contrast with the current idea of NLR activation, in which a DAMP or PAMP is needed to induce a conformational change and activate these proteins. It remains to be established if the LRRs of NLRC5 have PAMP or DAMP binding capacity that might further regulate activity. However, in the case of both NOD1 and NOD2, for which a clear activator has been defined, high expression levels are sufficient to confer autoactivation (65).

Although it has been presumed that plants and animals use different immune mechanisms to detect pathogens, there are interesting studies showing remarkable similarities in the structure and function of the receptors that recognize microbial antigens [reviewed by Ronald and Beutler (66)]. In fact, plants harbor Nucleotide-Binding-Leucine-Rich Repeat (NB-LRR) proteins, which have a similar structure to NLRs and also function as intracellular PRRs to ensure immunity against invading pathogens. Many of these NB-LRR proteins are present both in the cytoplasm and nucleus and function as transcriptional regulators [reviewed by Padmanabhan and Dinesh-Kumar (67)]. Recently, it was shown that two plant NB-LRR proteins [Barley mildew A (MLA) and *Nicotiana tabacum* TIR-NB-LRR immune receptor N] enhance pathogen-mediated defense gene transcription by forming a complex with specific plant transcription factors (68, 69). The similar function of CIITA and NLRC5 to these plant proteins makes it tempting to speculate that convergent evolution might also have selected similar regulatory mechanism (70).

## *In vivo* NLRC5 Function and Relevance in Health and Disease

### Lessons from knockout mouse models

The involvement of NLRC5 in MHC class I regulation was confirmed in *Nlrc5*-deficient mice by several independent studies (46, 51, 54–56). These works also enabled to determine the contribution of NLRC5 to MHC class I expression in different cell types. The greatest decrease in MHC class I expression was observed in CD4^+^ and CD8^+^ T cells, NK, and NKT cells; a significant defect was seen in B cells; and a more moderate defect in DCs and macrophages (46, 51, 54). The most prominent lack of MHC class I expression was therefore noticed among immune cells, where NLRC5 is mainly expressed at the steady-state (43, 46). However, analysis of MHC class I expression in thymic epithelial cells (TECs), revealed that *Nlrc5*-deficiency decreased their MHC class I display (46), indicating that NLRC5 also participates in MHC class I expression in non-hematopoietic tissues; a function that deserves further investigation.

Major-histocompatibility complex class I is not only the key molecule for CD8^+^ T cell activation and function but is also essential for thymic selection and peripheral maintenance of naïve CD8^+^ T cells. In line with that, two reports detected slightly reduced total CD8^+^ T cell levels in the spleen of *Nlrc5*-deficient mice under steady-state conditions (46, 56). This strongly suggests that the diminished MHC class I levels encountered in knockout animals affects CD8^+^ T cell homeostasis. In addition, the decreased surface MHC class I levels described above in *Nlrc5*-deficient TECs suggests that *Nlrc5*-deficiency might alter thymic selection and – potentially – the TCR repertoire.

The use of knockout animals also highlighted the ability of *Nlrc5*-deficient cells to increase their surface MHC class I levels following treatment with inflammatory stimuli such as IFNs or LPS, both in T cells and macrophages (46, 51, 54). In fact, although the defect in MHC class I expression observed in *Nlrc5*-deficient cells was maintained after stimulation, both control and knockout cells were able to substantially augment their MHC class I levels. This reiterates the existence of *Nlrc5*-independent mechanisms that regulate MHC class I (Figure 3).

### NLRC5 in infections

Given the recent discovery of NLRC5, we have just started to gain insight into the significance of this NLR in pathological conditions. Based on current knowledge one would have predicted it to be important in viral infections. However, the only information to date on the effects of *Nlrc5*-deficiency in this context come from an acute model of VSV viral infection in which no significant differences were observed between control and knockout mice (55). It would be extremely valuable to assess the ability of knockout animals to control less acute viral infections in which the adaptive immune response is activated.

Nonetheless, two studies clearly show that NLRC5 is important in the control of infection by *Listeria monocytogenes*, a Gram-positive motile bacterium that primarily infects monocytes and macrophages. One week post-inoculation, *Nlrc5*-deficient mice showed severely reduced numbers of IFN-γ-producing CD8^+^ T cells (51, 56). In agreement with this observation, bacterial clearance was affected and an increased bacterial burden observed in the liver and spleen of knockout animals. Surprisingly, the difference in restriction of *L. monocytogenes* infection was observed as early as 1 day after infection, indicating that besides the impaired CD8^+^ T cell-mediated response, an early defect in the innate response occurs in the absence of NLRC5 (56). The authors explained this by the observation of a partial defect in IL-1β production in *Nlrc5*-deficient mice upon infection. Future work aimed at dissecting the contribution of NLRC5 to the innate and the adaptive responses will be extremely important.

### NLRC5 and tumor surveillance

Because MHC class I surface expression was strongly diminished among *Nlrc5*-deficient lymphoid cells, the efficiency of CTLs in killing these cells was evaluated. In agreement with their reduced ability to present antigen, *Nlrc5*-deficient target T cells were eliminated less than control T cells by cognate CTLs *in vitro* (46). Although this observation still awaits confirmation *in vivo*, it has long been known that several malignancies, and in particular, hematological tumors, often lose MHC class I expression. This may enable more efficient evasion of immunosurveillance mechanisms. Mutations or epigenetic silencing that mostly target β2M or single HLA genes have been identified in these tumors (71). As such, NLRC5 mutation or silencing that results in downregulation of classical MHC class I in lymphoid malignancies could favor tumor development. Interestingly, the screening of a handful of lymphoid tumor cell lines suggested that NLRC5 is indeed expressed at low levels in several of these. In any case, NLRC5 expression level strongly correlates with the expression of a number of MHC class I genes in human tumor cell lines (Broad-Novartis Cancer Cell Line Encyclopedia). Future studies aimed at evaluating NLRC5 expression and mutations in a larger number of lymphoid tumor cells, and in primary tumors will contribute to delineate the potential importance of NLRC5 in tumor progression.

Displaying reduced MHC class I expression to escape CTL-mediated immunosurveillance can result in increased NK cell-mediated lysis. In fact, NK cells kill cells lacking the expression of MHC class I molecules, according to the “missing-self” hypothesis (72). For now studies addressing the role of NLRC5 in NK cell biology have yet to be performed.

## Emerging Functions of NLRC5 in Antiviral and Inflammatory Responses

The first characterization of NLRC5 showed that it can activate ISRE- and IFN-γ activating sequence (GAS)-containing promoters in human HeLaS3 cells (47). However, NLRC5 overexpression in other human cell lines failed to strongly activate important pro-inflammatory pathways such as NF-κB, mitogen-activated protein kinase (MAPK) or type I IFN (41, 43). Several studies reported that that overexpression of human NLRC5 actually resulted in an inhibition of NF-κB, activator protein 1 (AP1), and ISRE signaling; as well as TANK-binding kinase 1 (TBK1)- and SeV-mediated IFN-β promoter activation in HEK293T cells (41, 43, 45). The ability of NLRC5 to bind components of the NFκB and type I IFN pathway, namely IκB kinase (IKK)-α, IKK-β, retinoic acid-inducible gene 1 (RIG-I), and melanoma differentiation-associated protein 5 (MDA5) (45), led to the suggestion that NLRC5 overexpression interfered with these factors to inhibit inflammatory signaling. In spite of this, two independent studies using RNAi, revealed that human NLRC5 positively contributes to type I IFN expression upon viral infection in human cells (47, 53). This observation contrasts with the results of NLRC5 overexpression in HEK293T cells and highlights the difficulty in interpreting functional studies based on NLR overexpression (73).

In cells from *Nlrc5* knockout mice, PRR-mediated IFN and NF-κB signaling were also shown to be affected in embryonic fibroblasts, peritoneal macrophages, and – to a lesser extent – in BMDMs (55). However, no differences were observed in BMDMs and dendritic cells in complementary studies using three independently generated *Nlrc5* knockout mouse strains (46, 54, 74). In particular, work by Kumar and colleagues clearly showed that myeloid cells derived from *Nlrc5*-deficient mice showed no significant changes in the production of IFN-β or IL-6 after treatment with RNA viruses, DNA viruses, and bacteria (74). Moreover, serum IFN-β, IL-6, and IL-1β levels after polyI:C injection were also comparable between control and knockout mice. This discrepancy remains to be addressed by future works.

Many NLRs form multimeric complexes called inflammasomes that act as scaffold for the activation of the inflammatory caspase-1, which in turn cleaves and hence activates IL-1β and IL-18 (1). Several reports show that NLRC5 is able to enhance inflammasome-mediated IL-1β processing in human and murine cells. In human HEK293T cells ectopically expressing pro-IL-1β and pro-caspase-1, NLRC5 expression led to dose dependent IL-1β release (74). In isolated murine macrophages and dendritic cells derived from *Nlrc5*-deficient animals, activation of the NLRP3 and Aim-2 inflammasome was normal, although *Francisella tularensis*-mediated IL-1β release, that depends on Aim-2, might be affected (74). Also in human myeloid THP-1 cells, bacterial- and PAMP-induced IL-1β secretion, but not pore forming toxin-dependent activation of the inflammasome, was reduced upon silencing of NLRC5 expression (75). This was suggested to be mediated by interactions of NLRC5 with NLRP3 and the inflammasome adaptor protein ASC (56, 75). Despite the reported discrepancies, the observed effects of NLRC5 expression on the inflammasome and, more precisely, on NLRP3-specific stimuli deserve further research.

**Figure 4:**
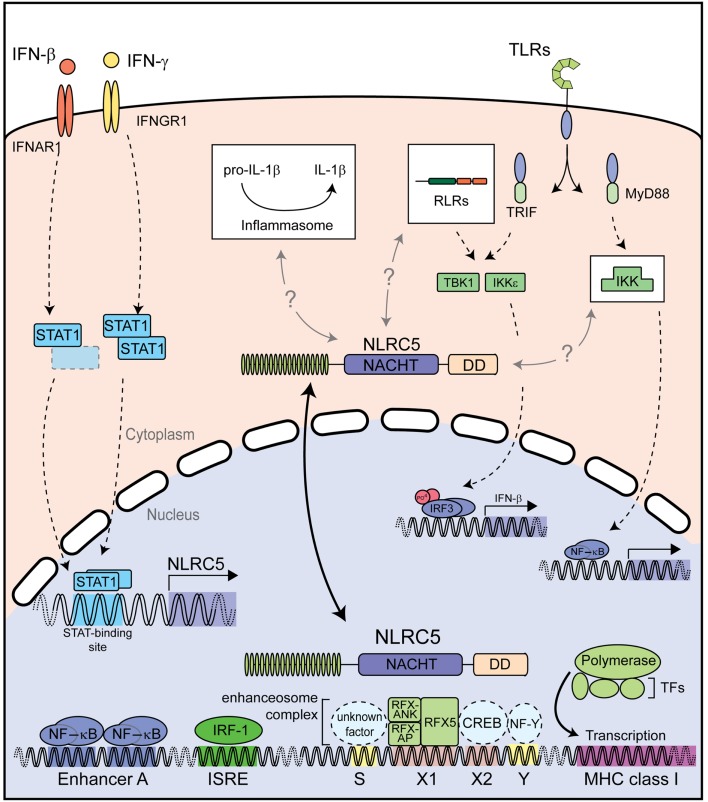
**Described functions of NLRC5**. Both type I and II IFNs strongly induce NLRC5 expression through STAT1 activation. NLRC5 can then shuttle into the nucleus and bind to the enhanceosome on MHC class I gene promoters, resulting in their expression. NLRC5 has also been shown to act as a modifier of NF-κB activity and type I interferon responses induced by PRRs recognizing microbial nucleic acids. Additional abbreviation: IRF, interferon regulator factor; ISRE, interferon stimulated response element; CREB, cAMP response element-binding protein; IKK, IκB kinase complex; IKKε, IκB kinase subunit epsilon; NF-Y, nuclear factor Y; RFX5, regulatory factor X 5; RFX-AP, RFX-associated protein; RFX-ANK, RFX-associated ankyrin-containing protein; RLRs, RIG-I-like receptors; TBK1, TANK-binding kinase 1; TFs, transcription factors; TLRs, toll-like receptors.

Taken together, NLRC5 appears to have distinct functions depending on its cytoplasmic or nuclear location (Figure 4). In the cytoplasm NLRC5 might influence canonical innate immune pathways, including type I IFN signaling and NF-κB signaling; at least in some cell types and species (41, 43, 45, 47). On the other hand, nuclear NLRC5 is a master regulator of MHC class I gene expression both in human and murine cells (42, 46, 53, 55, 56, 58). Additional studies are needed to elucidate the detailed cytoplasmic function of NLRC5, particularly with regard to the potential functional differences between humans and mice.

## Concluding Remarks

A substantial amount of evidence supports NLRC5 as the long sought after transcriptional regulator of MHC class I in human and murine cells, particularly in the hematopoietic lineage. However, emerging evidence suggests additional roles for this NLR in innate immune responses; roles we are only starting to understand and which require further investigation. We are beginning to gain insight into the molecular mechanisms underlying the transcriptional regulatory function and physiological relevance of NLRC5. In spite of the little understanding we have to date of NLRC5-mediated activities, its role as transcriptional regulator of MHC class I anticipates that important functions in health and disease await discovery.

## Conflict of Interest Statement

The authors declare that the research was conducted in the absence of any commercial or financial relationships that could be construed as a potential conflict of interest.
